# Should I Stay at Home Alone? Lived Experiences of Loneliness Among Older Adults: A Qualitative Study

**DOI:** 10.3390/healthcare13172101

**Published:** 2025-08-23

**Authors:** Maria Shuk Yu Hung, Michael Man Ho Li, Ken Hok Man Ho

**Affiliations:** 1 S.K. Yee School of Health Sciences, Saint Francis University, Hong Kong, China; 2 School of Nursing and Midwifery, La Trobe University, Melbourne, VIC 3086, Australia; k.ho@latrobe.edu.au

**Keywords:** older adults, loneliness, lived experience, Hong Kong

## Abstract

Background: Loneliness and social isolation among older people are currently widespread and recognized as the foremost public health problems globally and locally. Hong Kong, which exhibits a rapid aging trend and an expanding elderly population, is inevitably facing these issues. This study explored the lived experiences of loneliness among older adults in Hong Kong. Methods: Qualitative interviews were conducted among older adults in the community aged 60 or above who were cared for by migrant domestic workers and presented varying levels of loneliness. Purposive sampling was used to select subjects for face-to-face, semi-structured individual interviews, with consent for audio recording, which led to the inclusion of 19 older adults, among whom five were male, nine lived with a spouse, and three lived with their children. Interpretative phenomenological analysis was adopted. Results: We identified a core theme, “Should I stay at home alone?”, and the following four interrelated themes: (1) experience of inadequate social support and networks, (2) altered family dynamics and support, (3) deterioration in physical functions and mobility limitations, and (4) experience of negative and complex emotions. Conclusions: Based on our investigation into the lived experience of loneliness among older adults locally, we recommend that the government, non-governmental organizations, and healthcare institutions establish appropriate strategies and integrated services to address the social, physical, familial, and emotional issues in this population to foster healthy aging, improve their quality of life, and encourage support from families and communities.

## 1. Introduction

Life expectancy has increased globally [1], with one in six individuals aged 60 years or above in 2023. The World Health Organization emphasizes the importance of global collaboration for promoting healthy aging among older adults. Each country faces challenges in the expanding size and proportion of the older adult population, leading to increasing health and social service demands for older people. Apart from physiological changes and common health problems, aging is accompanied by other life transitions, including the death of friends and significant others [1]. People require social connections for personal development and to live a meaningful existence [2], as both social connections and networks have been shown to benefit physiological and psychological health [3].

At the other end of the spectrum from social connections is social isolation [4], found in individuals who lack meaningful connections or have a limited number of relationships [4]. Having a supportive social network and strong personal relationships is essential to feeling socially connected and reducing feelings of loneliness [4]. According to Weiss (1973) [5], loneliness is the individual’s perception of social isolation and being alone or disconnected from others, not just the absence of social contact; in the emotional sphere, it refers to the lack of intimate attachments and in the social one to the absence of a broader social network [5]. Loneliness is a common, subjective, unpleasant, and distressing experience that few people can completely prevent during their lifetime [5] and happens when a person’s social network is significantly lacking, either in quantity or quality [6]. Being lonely is often accompanied by feelings of emptiness and rejection [4]. Support from both kin and non-kin relationships can provide older adults with better protection against loneliness than support from kinships alone [4]. Moreover, relationships that lack support and involve conflict are often overlooked, yet they significantly impact an individual’s sense of loneliness. The convoy model of social relations suggests that individuals have various types of relationships with the people they are surrounded by, which creates a dynamic network significantly influencing their well-being [7].

Loneliness is a complex experience that can be affected by various factors rather than being a simple occurrence [4]. De Jong Gierveld’s multidimensional model of loneliness (1987) as a conceptual framework can explain the lived experiences of older adults holistically [8]. The model emphasizes that loneliness is a personal experience influenced by an individual’s perception and evaluation of social networks and connections that is articulated in three dimensions: emotional, social, and existential. Through this multidimensional approach, we can better understand the diverse experiences of loneliness among older adults, which aid in developing appropriate interventions to reduce their loneliness and enhance their overall well-being. Additionally, the findings of studies such as the present one, whose subjects are older adults assisted by live-in migrant domestic workers (MDWs), can guide the development of support services for older adults and provide relevant training for MDWs to better assist these individuals with their emotional and social needs.

Social isolation and loneliness can have a detrimental impact on well-being and quality of life [3], with growing evidence showing that they can exert adverse effects on the physical and psychological health, well-being, and quality of life of older adults in particular, including increased risk of stroke, heart problems, depression, cognitive decline, and even suicidal thoughts [3]. As people age, they are more susceptible to social isolation or loneliness because they are often alone [2]. Evidence shows that older Asian adults who lived in different countries suffered from loneliness and received insufficient social support [9]. In this population, loneliness and social isolation, exacerbated by the COVID-19 pandemic, are public policy and health issues requiring attention [3]. Su et al. (2023) conducted a systematic review and meta-analysis that examined the susceptibility of older adults to developing physiological illnesses and their propensity to experience mental health problems, particularly loneliness and social isolation, during the COVID-19 pandemic [10]; due to the high probability of loneliness (28.6%) and social isolation (31.2%), psychological interventions to improve this population’s psychological needs were recommended [10].

In this context, Hong Kong is a densely populated metropolitan city with a rapid aging trend and an expanding elderly population, where older adults aged 65 and over are estimated to reach 2.514 million (30.6% of the total population of 8.22 million) by 2043 [11]. Liu (2019) found that contributing factors for positive aging include perceived socio-financial conditions, family and peer social networks, social network magnitude, and emotional intimacy [12]. Better-quality dyad relationships and emotional intimacy with family and social support peers could facilitate positive aging in older people [12]. Although family support in traditional Chinese families considerably protects older adults from experiencing loneliness [13], different factors may influence family members’ or offspring’s readiness and feasibility to care for the well-being of the parents or elders, e.g., inadequate home caregivers, small home environments, low birth rates, and longer life expectancy [14]. Foreign domestic helpers (FDHs) have been vital to providing constant help and round-the-clock assistance to older adults globally, supplementing the declining support from family members [14,15,16,17,18,19,20]. Despite their functional support and assistance with daily tasks, it remains questionable whether their contributions can alleviate older adults’ experience of loneliness.

We conducted a large-scale mixed-methods study to investigate how the quality of dyadic relationships with MDWs is associated with older adults’ loneliness and the perceived care provided by MDWs [19,21]. The first phase of this study involved quantitative cross-sectional survey interviews of community older adults aged 60 and above who were cared for by live-in MDWs recruited from elderly community centers in Hong Kong for data collection and analysis [19,21]. A questionnaire administered during the survey interviews was used to collect the subjects’ demographic information and evaluate their functional status with the Lawton Instrumental Activities of Daily Living Scale [22], their emotional and social loneliness with the De Jong Gierveld Loneliness Scale [23], their relationships with their caregivers with the Mutuality Scale [24], and their social network and support with the Lubben Social Network Scale [25]. In the second phase, we additionally aimed to explore the lived experiences of lonely older adults cared for by MDWs; for this purpose, during the face-to-face survey interviews in the first phase, the 288 community older adult participants were asked whether they were willing to attend a second face-to-face individual in-depth interview if selected. In summary, this study presents the lived experiences of loneliness among older adults cared for by MDWs in Hong Kong. With a thorough understanding of older adults’ experiences of loneliness, relevant policies and appropriate interventions can be established to decrease feelings of loneliness in this population.

## 2. Materials and Methods

### 2.1. Design

The qualitative approach adopted in the second phase of this mixed-methods study followed the Consolidated Criteria for Reporting Qualitative Research (COREQ) [26].

### 2.2. Participants and Sampling

We used purposive sampling to recruit older adults at risk of loneliness, with low mutuality with migrant domestic workers, or experiencing low social engagement during the first interview using a questionnaire (Chinese version). These subjects had consented to participate and were selected and contacted by phone for the second phase of individual interviews. A sample size of about 20 was estimated to be necessary for data collection, or the latter was to be conducted until data saturation was achieved [27]. All the invited older adults participated in the interviews, conducted from mid-2022 to late 2023, without anyone withdrawing from the study.

### 2.3. Ethical Considerations

The Research Ethics Subcommittee granted ethical approval before this study’s implementation. The study information and purpose were explained to participants to ensure understanding, and their consent to participate and be audio-recorded was confirmed. The participants’ identities were maintained as anonymous, and their privacy and confidentiality were assured. The audio-taped interviews and transcripts were kept on an encrypted computer with restricted access, and all the data are to be destroyed five years after study completion.

### 2.4. Data Collection

One-to-one, in-depth, semi-structured interviews were used to collect data, which allowed the participants to describe their experiences in real time and flexibly [28]. The interviews were conducted in Cantonese (the common language in Hong Kong) by a researcher (the second or third author) with extensive experience in conducting face-to-face in-depth interviews; they were conducted in quiet rooms at the elderly community centers near the participants’ homes while their companions, i.e., their MDWs, waited outside to ensure privacy. The participants were encouraged to express their feelings, experiences, and challenges freely, and an interview guide, which included open-ended questions, was used to facilitate smooth implementation (Table 1). A few examples of the questions asked are as follows: “Based on your previous interview, you have a good/fair relationship with your MDW, but still feel lonely. Can you describe some of your daily interactions with your domestic worker?” and “What do you think about your loneliness, though you live with your family and MDW?” Follow-up and clarification questions were used accordingly. The interviewers carefully asked questions following a reflection on their preconceptions, avoiding using sensitive wording and facilitating the participants in expressing themselves. The participants’ emotional status was also closely observed, and a few participants were referred to the center counselor for further emotional and resource support with their consent. Before the interviews concluded, the information collected was summarized and verified with the participants. The total duration of all the interviews was 16 h and 50 min, averaging about 53 min per interview. The audio-recorded interviews were transcribed for analysis, and the field notes taken during and after the interviews were incorporated.

### 2.5. Data Analysis

Interpretative phenomenological analysis (IPA) was adopted for data analysis, as it allows for an in-depth exploration of how individuals perceive their lived experience [28,29] and focuses on details that comprehensively explain the individual case before seeking similar and different patterns across cases [30]. Rather than quantifying the data at hand, the purpose of IPA is to understand the crucial personal meaning of a participant’s life experience in a given context and situation [28]. Using IPA, researchers strive to comprehend and elucidate participants’ social, personal, and psychological worlds [28]. By studying the participants’ narratives, the research team attempted to reflect on and understand their perspective on life, especially their understanding of their current situation and relevant perceptions [29,31]. The researchers (the first and second authors) read through the transcribed interviews and individually noted down observations to identify repeated ideas and expressions; following a discussion between them, the authors further organized and categorized meaningful sections based on key features; then, they explored themes and subthemes derived from the transcription to identify individual participants’ similarities and natural variations, linking the themes and determining relationships within the data. Data saturation was reached after 19 participants had been interviewed. Finally, the researchers further interpreted and refined the main themes and subthemes.

## 3. Findings

Among nineteen older adults cared for by live-in MDWs who were at risk of loneliness and social isolation, five were male, one was single, nine were widowed, nine lived with a spouse, and four lived with their children, and their ages ranged from 67 to 94. Table 2 presents the demographic information and living status of the participants.

Regarding the lived experiences of loneliness among older adults cared for by live-in MDWs, a core theme emerged, alongside four interconnected themes, which illustrate how participants perceive their experiences of loneliness. The core theme was “should I stay at home alone?”, and the four interconnected themes of physical, social, familial, and emotional aspects identified were (1) deterioration in physical functions and mobility limitations, (2) experience of inadequate social support and networks, (3) altered family dynamics and support, and (4) experience of negative and complex emotions, respectively. The core theme, the interconnected themes, and the subthemes are shown in Table 3.

The core theme, “Should I stay at home alone?”, highlights the reluctance of older adults to be left alone. They expressed a desire not to be left alone or socially isolated; however, physical, social, familial, and emotional factors often prevented the fulfillment of this desire. The concept of “home alone” extends beyond its literal physical meaning, which is often influenced by bodily dysfunctions or disabilities that limit mobility; it also encompasses indirect barriers such as insufficient social support, environmental constraints, altered family dynamics, and complex emotional experiences. These factors contribute to older adults spending more time at home or being left alone, which can exacerbate loneliness and social isolation.

### 3.1. Deterioration in Physical Functions and Mobility Limitations (Physical)

The first theme, “deterioration in physical functions and mobility limitations,” comprises two subthemes, the fear of falls and injury and the inability to go on outings independently, and relates to the participants’ safety, pre-existing health problems, and the normal aging process, which impacts personal health and mobility, all common causes of concerns among our participants. Most of them had more than one chronic illness, which hindered their everyday life and social activities; moreover, coronavirus infection had affected the health of some participants, further exacerbating their condition.

#### 3.1.1. Fear of Falls and Injury

Due to a decline in physical and cognitive functioning, some participants expressed concerns about falls and potential injuries. Staying at home was preferable and made them feel safer, particularly when accompanied by their “maid” (domestic worker). One single female participant had vision impairment and resided in a place with few residents. She narrated her fear and loneliness as follows:

*I reside in an old estate, and most citizens have moved out. It’s so quiet, and the surroundings are dim at night. I am scared and feel lonely as no one is with me. My vision has worsened in recent years, and I cannot see things clearly. Even when I go out, I cannot see or be aware of the oncoming vehicles*

(P13).

An 83-year-old widow talked about her physical health deterioration after getting infected during the COVID-19 pandemic and her fear of falls since then. She felt helpless and described her dizziness as so sudden that she could not be prepared or controlled, as follows:

*After I recovered from coronavirus, my health deteriorated. One time, I lost consciousness and fell outside. I was admitted to the hospital for treatment. Since then, I have had sudden vertigo from time to time, and the surroundings seem to be moving around. I was so scared I had to ask someone to take me home immediately. After that, I stayed home mostly with my maid. I wish I could be healthy and free to go out*

(P15).

#### 3.1.2. Inability to Go on Outings

While some participants were hesitant to go on outings, others wished to go out as usual. Unfortunately, a few could not go out alone because they were unable to walk steadily or adequately, and their bodies could not tolerate prolonged outdoor activities due to mobility limitations. A 94-year-old lady who lived with her husband and MDW enjoyed traveling outside Hong Kong with her close friends when she was young; however, due to severe back pain and mobility problems, she claimed she had no choice but to stay home. She verbalized her loneliness and disappointment as follows:

*We (and her husband) planned to travel elsewhere after retirement… However, our health is not so good. He had various chronic illnesses, including cancer, and had undergone an operation. Same to me, my severe back pain improved a bit after surgery, I could only walk for a while… We cannot go traveling anymore…*

(P1).

A 70-year-old lady had a stroke and has lived with her husband and her domestic worker in recent years; following the stroke, she was frustrated about her body weakness, slurring, and related symptoms that decreased her mobility.

*I have participated in various group activities at the community center before, such as handicraft and singing classes. After the stroke, I was unable to join or enjoy the classes because of the affected body and hand movements. I stay home mainly… Sometimes, the maid accompanies me for a walk or shopping…*

(P19).

No matter whether the participants’ staying home was related to their safety concerns or inability to go out, they suffered from moderate to severe loneliness due to deteriorating physical functions and mobility limitations. Despite the MDWs accompanying them on outings for exercises or activities, they could not participate in or enjoy the activities as they had before.

### 3.2. Experience of Inadequate Social Support and Networks (Social)

The second theme, “experience of inadequate social support and networks,” relates to the social aspects of superficial relationships among neighbors and friends, as well as diminished social support and social activities. As they aged, some participants experienced reduced social interactions, support, and networking with others, such as their close friends or relatives. Furthermore, the negative impacts of the COVID-19 pandemic over the past few years also reduced social activities and gatherings.

#### 3.2.1. Superficial Relationships with Neighbors and Social Friends

Although some participants established good relationships with their neighbors, others reported that only superficial relationships were maintained with neighbors and friends in the elderly community centers, which differed from the relationships they had with their old friends. Indeed, trust and sharing are essential to establishing long-term social support relationships. One female participant lived with her husband and differentiated between the relationships she had with her old friends and neighbors.

*I had old friends with whom I had a good relationship and shared happiness, but they were dead. I have good neighbors, but the relationship is not deep. We meet during our morning exercise. We chat casually, but we’re not particularly close*

(P1).

A widower participant who visited the elderly community center on weekdays commented on his relationships with friends there as follows:

*They are only social friends, not close friends. Spending the day with them in the center is better than staying home alone. Just chatting, watching TV, having lunch together…*

(P3).

#### 3.2.2. Diminished Social Support and Social Activities

With the participants getting older, their social networks and connections had shrunk due to their close friends having died of medical illness in the past few years or them having fewer contacts with their friends after retirement. Additionally, various interconnected factors, such as physical incapacity or social distancing measures, may have further impacted the participants’ involvement in social activities. A retired taxi driver aged 89, who lived with his wife and domestic worker, shared his reduced social connections:

*I had work colleagues, good friends, and social friends when I was young. You know, after retirement and getting older, we seldom met and contacted each other… They passed away one by one, and even my relatives became fewer. It’s life… It’s inevitable…*

(P8).

Another participant, aged 83, echoed this sentiment and sorrowfully spoke of her friends’ and relatives’ deaths:

*Most of my close friends have passed away. Early this year (in 2022), my younger sister got infected with the coronavirus and was admitted to the hospital. Just three days later, she passed away. Then, her husband (brother-in-law) was also infected and passed away three weeks later. You know, it’s so sudden and unexpected*

(P4).

Apart from the death of old friends, other participants claimed that their poor health made them distant from social friends and activities.

*We have frequently visited Shenzhen (a Chinese city near Hong Kong). Now, due to my leg problem, the pandemic social distancing measures before, and my close friends passed away, no more tours or trips*

(P17).

Different interrelated factors, such as physical deterioration, environmental constraints, the death of close friends, and personal health problems, affected the participants’ social support and networks, leading to a decrease in social activities and gatherings to a certain extent.

### 3.3. Altered Family Dynamics and Support (Familial)

The third theme, “altered family dynamics and support,” concerns intimate familial connections, interactions, care, and support among family members. The three identified subthemes are strained family relationships, the loss of family support, and families’ unavailability for in-person visits. Family dynamics refer to the complex interactions and relationships among family members, which can be influenced by various factors, including traditional beliefs. Traditional beliefs regarding home unity and harmonious living are deeply rooted in the minds of most older-generation individuals in China and Hong Kong.

#### 3.3.1. Strained Family Relationships

Even though some participants reported having good or satisfactory relationships with their children, others had strained or poor relationships with their spouses or children, regardless of whether they lived with them. The following two participants experienced strained family relationships, including conflict with or neglect by their children, which led to estrangement among family members or the avoidance of unnecessary communication.

*My husband passed away seven years ago, and then I lived with my daughter and son-in-law. I had a previous quarrel with them. Since then, they seldom talked to me, especially after she had employed a migrant worker. She only communicates with the maid but neglects me. I don’t understand what the maid says, as she is Indonesian (using a different language). My son last visited me a year ago*

(P5).

The aforementioned older woman, aged 90, was unable to walk. Although she had a large family spanning four generations, her two sons and their families rarely visited or contacted her, and she had no other relatives in Hong Kong. Relationships that lack support and involve conflict can considerably affect an individual’s sense of loneliness. Another widow of the same age, who had a strained relationship with her children, expressed a similar situation:

*My husband died many years ago. I’ve lived alone for 8 years, and my health has not been good in recent years. I’ve two daughters and two sons, but the relationships are not good… I live with my elder daughter, her husband, and an Indonesian maid. Others seldom contact me. We had argued about the property issue…*

(P7).

#### 3.3.2. Loss of Family Support

Some participants reported a lack of family support, often due to the death of a spouse or not living with their children. Of the nine widows and widowers, only three lived with their children. One widow, aged 87, expressed a desire for physical and practical assistance from her children, particularly during times of illness:

*I have three children. My daughter is mentally incompetent and lives in a residential care home. The son is a teacher, and the other daughter is a nurse. They live and work in distant places, visiting me once a month. When I was sick, I would call them. I would be happy if they could come frequently and join me for dinner*

(P18).

Some participants received physical support and/or financial support from their children, including assistance with attending medical appointments or paying for domestic workers, respectively. Sadly, an 85-year-old widower recounted his experience of lacking physical, emotional, and financial support from his children for more than twenty years:

*I have lived alone for more than twenty years after my wife’s death. Even though I was sick, no one helped or cared about me. I’m old. They (the children) seldom contact me, especially after I employed a maid to care for me two years ago. We (with the maid) use body language to communicate, as she speaks English. I am unhappy and feel lonely… I have no one to talk to*

(P6).

#### 3.3.3. Family’s Unavailability for In-Person Visits

Most participants expressed a desire for more frequent visits from their children. While some were visited regularly (about once a month) or occasionally (around once a year), they still experienced feelings of loneliness. In addition to being busy or living far away locally, some participants mentioned that their children were unavailable for in-person visits after emigrating to other countries.

*I have two sons and two daughters. My elder son and daughter emigrated to England and Australia and seldom contact me. Another daughter provides financial support, and the younger son lives far away and occasionally visits us (and her husband) for my husband’s hospital follow-up appointments and performs house maintenance*

(P11).

*My husband passed away years ago. We have three sons, one of whom has been living in Ireland for over 30 years. One stayed in mainland China mostly, and the other lives in Hong Kong. Sometimes I miss them so much and feel a deep sense of loneliness… Though they were not living with me, they employed the maid to care for me*

(P17).

The narratives indicated that family dynamics and support were not influenced by whether the participants lived with their children. However, the cultural belief of filial piety—emphasizing the importance of caring for parents—was deeply rooted in the participants’ discourse. They expected to receive support from their children, whether in the form of physical, psychological, or financial assistance. Filial piety and support could manifest in various ways, such as hiring domestic workers for financial aid, accompanying parents to medical appointments for physical support, and making frequent video calls or visits for psychological support, which may decrease feelings of loneliness.

### 3.4. Experience of Negative and Complex Emotions (Emotional)

The last interrelated theme, “Experience of negative and complex emotions,” reflects the older adults’ personal psychological and emotional states and consists of two subthemes concerning their experienced helplessness and feelings of emptiness. These negative and complex adverse feelings are closely connected with other social, familial, and physical impacts.

#### 3.4.1. Experienced Helplessness

Due to their declining physical health, reduced social activities, and familial changes, the participants experienced helplessness and frustration in daily life, especially when they perceived that they could receive help from or rely on nobody. While the MDWs could offer assistance with daily activities, their support was not the same as that of their families and was not always successful for several reasons. One participant, aged 83, lived with his wife and had no children. After his wife had a stroke, he employed a domestic worker to assist in caring for her. He sadly articulated his worries about their health and the future:

*We are old, and she (his wife) has been paralyzed for years after a stroke. She had an awful experience residing in a private nursing home for a few months. Then, I hired and changed a few maids to assist me in daily housework and caring for her. I teach the maid Cantonese so that the maid can communicate with her. I’m older than her (his wife), and I hope to live longer to care for her. I cannot imagine what her life would be without me…*

(P14).

Another participant, with poor physical health and vision, voiced her difficulties in daily living and her feelings of helplessness:

*I am not married and do not have any children. My sister-in-law has hired a migrant worker to take care of me. She accompanies me to the hospital. However, she will be leaving in November. My sister-in-law may be unable (financially) to hire a new maid for me. I am so worried… What can I do?*

(P13).

#### 3.4.2. Feelings of Emptiness

Apart from helplessness experiences, some participants had feelings of emptiness related to familial changes and physical illness. Their children moving out of the family locally or overseas first sparked their sense of emotional emptiness and loneliness. Additionally, physical diseases, especially those requiring surgical operation, triggered the participants’ feelings of physical emptiness. One widow, aged 83, had a distant relationship with her children and only lived with her MDW; she expressed her fear and emotional emptiness as follows:

*My husband died years ago, and my children are far away (emigrated)…I wish they could be with me… I told myself to accept their decision… I have no choice, and my life has changed… I do not enjoy video calls… I’m so weak, and seldom go out. My health is worsening, and I was admitted to the hospital before… They (the children) employed a maid for me*

(P15).

Another widowed participant, aged 87, did not live with her children, who visited her once a month and with whom she maintained a fair relationship, and had undergone multiple surgical operations for her physiological illnesses. She described her feelings and fear of death as follows:

*I often feel unwell and experience discomfort. I have multiple health issues and have undergone several surgeries, including the removal of my breast, gallbladder, and uterus. I have also had procedures for cataracts, glaucoma, and a corneal transplant. I have a brain tumor, but I informed my doctor that I prefer not to have surgery anymore… I have an empty body only… I’m scared that I may die soon*

(P18).

The narratives reflected that these participants experienced different complex emotions, such as helplessness, hopelessness, emptiness, and fear of death. They perceived that they had no choice but to accept changes in their health and familial situations, which further worsened their well-being and mental health.

## 4. Discussion

This study’s findings demonstrate that various factors, including physical, social, familial, and emotional ones, contribute to the loneliness experienced by older adults. Though the study participants were under the care of MDWs and some lived with family, they still perceived social and emotional loneliness. Consistent with a local study, social and emotional support from family and peers (such as friends, neighbors, and community center affiliates) is crucial to the elderly community [12].

In Hong Kong, it is forecasted that individuals aged 65 and above will exceed 2.5 million by 2043, accounting for over 30% of the total population [11]. The rapidly aging population increases the demand for comprehensive and high-quality health and social services to promote healthy aging among citizens locally. Older people usually have one or more chronic illnesses and require considerable support and care, as common health problems such as hearing deficit, vision loss, chronic pain, respiratory diseases, depression, and dementia, similar to what our study participants had, commonly occur in older age [1]. The WHO indicates that supportive physical and social environments are crucial to facilitating older people in performing activities that are valuable to them, regardless of deterioration in physiological capacity [1]. In addition to domestic workers’ company and assistance, which relieve older adults’ anxiety while on local outings, providing safe and accessible public facilities including buildings and transport and ensuring access to low-difficulty walking paths are supportive measures for aging individuals that might facilitate them in going on outings despite their physical limitations, improve their social connections, and decrease their social loneliness.

Apart from physiological and cognitive deterioration related to aging, inadequate social support and networking are other factors contributing to the study participants’ loneliness, particularly in relation to the adverse health and social impacts caused by the COVID-19 pandemic over the past few years. The local social distancing policies and measures implemented considerably disrupted their social support and networking with others, and the death of close friends and the temporary closure or limited services of the elderly community center further reduced their support and social activities. Evidence has shown that implementing public health measures during the pandemic increased the general public’s loneliness and social isolation globally, particularly among older adults [10]. Su et al. (2023) reviewed 30 studies of 28,050 older adults from 15 countries in Europe, North and South America, and Asia [10], and reported a 28.6% pooled prevalence of loneliness during the pandemic period and a 31.2% prevalence of social isolation, with higher values in studies after three months of the pandemic than in those before this time point. The global implementation of social distancing policies, which aggregate obstacles to connecting with others, may be a reason for the elevated prevalence [10], aligning with our study findings in Hong Kong [21,32]. Indeed, maintaining physical distancing but engaging in social connection is essential [33]. Wu (2020) suggested addressing older adults’ loneliness and social isolation by utilizing community-based networks and resources provided by family members [33].

Compared with the previous SARS and Swine Flu outbreaks, the unexpectedly long duration of the COVID-19 pandemic has led to more severe, long-term social, financial, physical, and psychological health impacts on local populations [34,35,36,37,38] and older individuals [21,39]. Due to the adverse effects on the health and well-being of the latter, the WHO (2021) recommended effective strategies and interventions at various levels, including individual and relational, community, and social levels, to mitigate social loneliness and isolation among older people [3]. The authors of a review examined the similarities and differences in how various countries manage loneliness and suggested that policymakers should concentrate on several key areas: using effective language, prioritizing interventions, revisiting past activities, sharing successful strategies across countries, establishing a clear vision, evaluating the effectiveness of interventions, and ensuring that these interventions can be implemented quickly and sustainably [40]. Additionally, a recent review analyzed studies conducted between 2003 and 2023 that focused on interprofessional collaboration in various interventions aimed at helping individuals experiencing loneliness [41]; these interventions included social groups, social skill training, art therapy, home visits, intergenerational support, and coordinated care pathways and, overall, were found to be effective in reducing loneliness among older adults. Therefore, it is recommended that relevant policies and appropriate programs be developed locally to decrease social isolation and loneliness among older adults.

In addition to inadequate social support, family dynamics and support are crucial determinants of health and well-being in older adults [42]. Unhealthy family dynamics, including conflict or neglect, may lead to adverse health consequences and psychological impacts. The death of spouses or family members and the reduction in family members’ visits aimed at minimizing cross-infection during the pandemic also lessened family support, which might have further contributed to the loneliness in older adults’ observed in our study. According to traditional Chinese family ethics, a family fosters close bonding, cultural filial piety, and respect for the elders and encourages family members to live together, promoting intergenerational support, which is crucial to maintaining family harmony and stability [43]. Our findings showed that most participants retained traditional beliefs, incorporating familial concepts of family obligations and responsibilities, and wished to live with their children and maintain family harmony. The findings align with the previous literature highlighting the rapidly aging population and family members’ emigration to other countries as important factors influencing conventional family structural changes and beliefs [44]. The offspring emigrate overseas, but their parents are left behind in Hong Kong; the weakening of intergenerational care and support is unavoidable, with parents and elders being thus denied the filial piety aspect of being looked after that is traditional in Chinese culture [43,45]. This may be a factor that exacerbates the emotional loneliness experienced by older adults. Even though a better relationship between older adults and MDWs may have a positive influence on the loneliness of the former [19], family bonding and relationships cannot be easily compensated or replaced by MDWs. Indeed, family is the fundamental source of support and can enhance the mental health and subjective well-being of Chinese older adults [46]. Our study participants’ social network and the support they received from family may have been weakened due to their offspring’s emigration, which further increases loneliness in Chinese older adults [46].

According to local statistics, 113,200 Hong Kong residents left between mid-2021 and mid-2022 [47]. Our study participants’ offspring’s migration decisions resulted in the latter’s unavailability for in-person family visits, which further aggravated the loneliness of older adults, echoing the results of other local studies [48,49]. The Hong Kong Christian Service [48], in early 2023, surveyed 203 elders whose offspring had emigrated in or after 2020 and reported that 79.5%, 69.9%, and 69.9% of participants were at high risk of social isolation, loneliness, and susceptibility to depression, respectively. Consistent with our study findings, children’s emigration has an impact on the lifestyle, role perception, and emotional well-being of the left-behind parents [49]. These studies suggested establishing a “Neighbourhood Support Network” and using information technology to help older adults stay connected with their emigrant children [48,49].

Apart from family, friends, neighbors, government/social organizations are also considered sources of support in the convoy model of social support for individuals [46]. Chen et al. (2014) reviewed evidence that support from friends, including the number of friends with whom older adults maintain regular contact, the number of close friends they have, and the frequency of interaction with these friends, is inversely related to psychological health issues and positively associated with mental well-being [46]. Though similar support was found for our participants according to their narratives, some of them stated that their close friends were dead or had decreased contact, especially during the pandemic, which might explain the inadequate social support they received from friends. Furthermore, our study participants maintained only superficial relationships with their neighbors, which also reflected the limited support they received from them.

Today, an increasing number of older adults own smartphones or personal computers and utilize mobile technology to communicate with friends and family, as well as for accessing resources and information [33,45]. Social media software applications, such as Skype, Zoom, WhatsApp, and WeChat, help connect family members who are far away and reduce the adverse impacts of social isolation and loneliness [33,45]. However, these applications cannot fully replace their physical and psychological connections, particularly for those among the elderly who are incapable of using these devices or are unfamiliar with them and cannot enjoy any kind of technological linkage. This may further disconnect them from their family members or exacerbate their feelings of loneliness or social isolation [45], aligning with the narratives of our participants who lived alone with MDWs. MDWs might lessen the workload of daily domestic affairs but cannot fully alleviate feelings of loneliness and compensate for the lack of psychological support from family members in some older adults. There is a growing need for social support for older parents who are left behind in Hong Kong or do not live with their children.

The deterioration in physiological health among the aging population, the emigration of siblings and younger generations, and the adverse impacts of the COVID-19 pandemic have given rise to various physical, psychological, familial, and social health issues and life challenges, leading to loneliness and social isolation among older adults in Hong Kong. The local government, non-governmental organizations, and healthcare institutions are recommended to formulate relevant policies and implement appropriate service programs to provide greater support to those who might be neglected and experiencing loneliness and social isolation. For instance, further measures apart from existing elderly care centers and services could include more community home visits by professional teams to provide physical care, emotional counseling, assistance with the development of mobile technological skills, and social support, especially for those who do not live with their families and present physical problems that limit their mobility, are homebound, or are socially isolated. We recommend developing appropriate strategies with a collaborative approach and providing integrated services to address the social, physical, familial, and emotional needs of older adults, fostering healthy aging, improving quality of life, and encouraging family and community support in this population.

This study has limitations. The participants were selected from a pool of 288 Chinese older adults in 34 elderly community centers of different districts and invited purposefully to participate in a face-to-face survey interview in the first study phase; as these older adults were referred by the elderly centers that joined this study, they may have been more active in participating in social activities and may have had better social networks. Another limitation lies in the adopted interpretive phenomenological approach, which, rather than quantifying the data, aims to understand the crucial personal meaning of a participant’s life experience in a given context and situation [19]; however, with this methodology, the researchers aimed to comprehend and elucidate the social, personal, and psychological worlds of the participants, as carefully identifying subjects with wide variations and information-rich data can facilitate a more in-depth understanding [50].

## 5. Conclusions

Various contributing and contextual factors may exacerbate loneliness and social isolation in older adults. On the basis of our investigation into the lived experience of loneliness among older adults locally, we recommend that the government, non-governmental organizations, and healthcare institutions establish appropriate strategies and integrated services to address the social, physical, familial, and emotional issues in this population to foster healthy aging, improve their quality of life, and encourage support from families and communities.

## Figures and Tables

**Table 1:** Interview guiding questions.

**Examples of Open-Ended Guiding Questions.**
Based on your previous survey interview data, you have a good/fair/poor relationship with your domestic worker, and you also experienced a mild/moderate/intense sense of loneliness. Can you describe some of your daily interactions with your domestic worker? What are your thoughts on your relationship with your migrant domestic worker? Regarding your living situation, you live with a domestic worker and your family/but not with your family. Can you share some of your interactions or communications with your family members? How do you feel about your relationships with your family members, such as your children or partner? Despite living with a migrant domestic worker (and your family), how do you perceive your loneliness? Can you share some of the social and daily activities you have participated in over the past few months or years? What are your thoughts on your relationships with your friends and neighbors? How do you feel about your current health condition?

**Table 2:** The demographic information and living status of the older adult participants.

No.	Gender	Age	Marital Status	Living with Family
1	Female	94	Married	Husband
2	Female	84	Married	Husband
3	Male	73	Widowed	---
4	Female	83	Married	Husband
5	Female	86	Widowed	Daughter and son-in-law
6	Male	85	Widowed	---
7	Female	90	Widowed	Daughter and son-in-law
8	Male	89	Married	Wife
9	Female	80	Widowed	Daughter
10	Female	75	Married	Husband, son, daughter-in-law, and grandson
11	Female	87	Married	Husband
12	Female	67	Married	Husband
13	Female	75	Single	---
14	Male	83	Married	Wife
15	Female	83	Widowed	---
16	Male	84	Widowed	---
17	Female	90	Widowed	---
18	Female	87	Widowed	---
19	Female	70	Married	Husband

**Table 3:** The core theme, the four interconnected themes, and the subthemes.

Core Theme	Interconnected Themes	Subthemes
Should I stay at home alone?	Physical— Deterioration in physical functions and mobility limitations	Fear of falls and injury Inability to go on outings
	Social— Experience of inadequate social support and networks	Superficial relationships among neighbors and social friends Diminished social support and social activities
	Familial— Altered family dynamics and support	Strained family relationships Loss of family support Family’s unavailability for in-person visits
	Emotional— Experience of negative and complex emotions	Experienced helplessness Feelings of emptiness (physical and emotional)

## Data Availability

The data presented in this study are available upon reasonable request from the corresponding author. They are not publicly available due to privacy or ethical restrictions.
